# The Effect of Introducing B and N on Pyrolysis Process of High Ortho Novolac Resin

**DOI:** 10.3390/polym8030035

**Published:** 2016-02-24

**Authors:** Jin Yun, Lixin Chen, Xiaofei Zhang, Junjun Feng, Linlin Liu

**Affiliations:** 1 Department of Applied Chemistry, School of Science, Northwestern Polytechnical University, Xi’an 710072, China; yunjin1988@126.com (J.Y.); 987595067@mail.nwpu.edu.cn (X.Z.); fengjunjun1182@163.com (J.F.); 2 Science and Technology on Combustion, Internal Flow and Thermal-Structure Laboratory, School of Astronautics, Northwestern Polytechnical University, Xi’an 710072, China; viola7788521@163.com

**Keywords:** phenylboronic acid, novolac resin, thermal stability, chemical state

## Abstract

In this contribution, high ortho novolac resins modified with phenylboronic acid were synthesized. The thermal stability of novolac resins cured with hexamethylenetetramine (HMTA) and chemical states of B and N via a pyrolysis process were studied. For the cured *o*-novolac modified with phenylboronic acid, the temperature with maximum decomposition rate increased by 43.5 °C, and the char yield increased by 5.3% at 800 °C compared with cured *o*-novolac. Density functional theory (DFT) calculations show the existence of hydrogen bonding between N of HMTA and H of phenol in modified resin. Thus, N could still be found at high temperature and C=N structure could be formed via a pyrolysis process. B_2_O_3_ was obtained at 400 °C by the cleavage of B–O–C and B–C bonds and it reduces the oxygen loss which may take part in the formation of carbon oxides in the system. The melting B_2_O_3_ on the surface of the resin will prevent small molecules and carbon oxides from releasing. Moreover, introducing B into the system helps to decrease the interlayer distance and improve graphite structures via a pyrolysis process.

## 1. Introduction

Phenolic resins (PF) have been used since 1907 for a wide variety of applications such as thermal insulation materials, molding powders, laminating resins, adhesives, binders, surface coatings, and the matrix for carbon composite materials, because of its low cost, excellent ablative properties, and thermal stability [1,2,3,4,5,6,7,8,9]. However, PF was not able to completely meet the constantly developing requirements which most focus on the thermal properties [10]. To improve the thermal properties of PF , the addition of phosphorus, boron, and silicon compounds has been used [11]. Introducing boric compounds, such as B_4_C [12,13,14,15], boric acid, phenylboronic acid [16], BN [17], into resin to synthesize boron-modified resin (BPF) is one of the most successful ways to modify phenolic resin. Gao *et al.* prepared BPF by using boronic acid as boron source and base as catalyst and pointed out that there were two way to react in the system, the reactivity of boronic acid with hydroxymethyl groups was much higher than that of phenolic hydroxyl [18,19]. Wang *et al.* introduced boronic acid and phenylboronic acid into resoles to synthesized BPF [16]. However, there is little published research about boron-containing high ortho novolac resin (B-*o*-novolac), as well as their pyrolysis mechanisms. 

Compared with conventional resoles, high ortho novolac resin (*o*-novolac) exhibits a much more rapid curing behavior with hexamethylenetetramine (HMTA) and novolac resin has lower viscosity [20,21]. Moreover, phenylboronic acid has one aromatic ring which can improve the thermal property and reduce the possibility of gelling when synthesized with *o*-novolac [22].

In this work, we synthesized B-*o*-novolac by introducing phenylboronic acid into novolac resins and analyzed the structure and thermal properties of cured B-*o*-novolac, comprehensively. In addition, we also evaluated the chemical state changes of B and N in pyrolysis process and their effects on char yields.

## 2. Experimental Section

### 2.1. Materials

Phenol, 37% aqueous formaldehyde, organic acid of bivalent metal salt catalyst (Catalyst I), sodium hydroxide, methyl isobutyl ketone, HMTA, ethanol were purchased from Tianjin Chemical Reagent Co., Tianjin, China. phenylboronic acid (99.8%) was received from Beijing Huawei-Ruike (HWRK) Chem. Co., Ltd. (Beijing, China). All materials were of analytical grade and used as received without further purification.

### 2.2. Synthesis of B-o-novolacs

Phenol (40.00 g), formaldehyde (24.15 g), and Catalyst I (0.80 g) were added into 250 mL three-necked flask equipped with a stirrer, a cooling condenser, and a thermometer. The system was slowly heated using an oil bath and reacted at 100 °C for 4 h. Then the water was extracted. After that, sodium hydroxide (0.40 g) and phenylboronic acid were added into the system and reacted for 4 h at 140 °C. The molar ratio of phenol to phenylboronic acid was 1:0.35~1:0.20 (B_0.20_-*o*-novolac: 1:0.20; B_0.25_-*o*-novolac: 1:0.25; B_0.30_-*o*-novolac: 1:0.30; B_0.35_-*o*-novolac: 1:0.35). After that, methyl isobutyl ketone (60.00 g) was used to extract water and catalysts. Finally, subjecting the solution to a vacuum at 120 °C until the total extraction of water and solvent was completed.

### 2.3. Synthesis of o-Novolac

Adding the same dosage of phenol, formaldehyde, and Catalyst I into 250 mL three-necked flask equipped with a stirrer, a cooling condenser, and a thermometer. The system was slowly heated using an oil bath and reacted at 100 °C for 8 h. Then methyl isobutyl ketone was used to extract water and catalysts. Finally, subjecting the solution to a vacuum at 120 °C until the total extraction of water and solvent was completed.

### 2.4. Preparation of the Cured B-o-novolac

Blend sample was prepared by mixing B-*o*-novolac with 12% HMTA in ethanol under magnetic stirring at 50 °C for 2 h until a homogenous solution was obtained. Then, the solvent was evaporated at 60 °C under vacuum. The concentrated sample was cured in a vacuum oven at 135 °C for 2 h, 150 °C for 2 h, and 170 °C for 4 h.

### 2.5. Preparation of Carbonized Samples

To analyze the chemical state of B and N in pyrolysis process, about 2.0 g cured B_0.30_-*o*-novolac or cured *o*-novolac were placed in graphite crucible and heated from room temperature to the targeted temperature for 2 h with a heating rate of 10 °C/min in Ar. The cured resins were carbonized at 200 °C/2 h, 400 °C/2 h, 600 °C/2 h, 800 °C/2 h, 1000 °C/2 h, and 1200 °C/2 h, respectively. The tube furnace was cooled from targeted temperature to room temperature with a rate of 5 °C/min.

### 2.6. Characterization

The Fourier transform infrared (FT-IR) spectra were recorded with a width of 4000–500 cm^−1^ to confirm the structure of the B-*o*-novolacs and *o*-novolac by using FT-IR spectrometer (BRUKER, Ettlingen, Germany). 

The ^11^B nuclear magnetic resonance (NMR) spectra were obtained using a Bruker Avance 400 MHz apparatus in dimethylsulfoxid-d6.

DEPT-135 NMR spectra were recorded on a Bruker Avence (Bruker, Fällanden, Switzerland) 400 MHz spectrometer with tetramethylsilane (TMS) as an internal standard and deuterated dimethylsulfoxide-d6 as solvent.

The thermalgravimetric analysis (TGA) of the cured resins were conducted on a thermogravimetric analyzer Q600SDT (TA, New Castle, USA) under dry Ar gas. The relative mass loss of the samples was recorded from 25 to 1200 °C with speed of 10 °C/min and the carbon yields of cured resins were gotten.

The X-ray photoelectron spectroscopic (XPS) tests were carried out using a K-alpha spectrometer (Thermo Fisher Scientific, Waltham, MA, USA) and the core level spectra were measured using a monochromatic Al K R X-ray source. Binding energies were referenced to the C1s peak at 284.80 eV and the curve fitting of the XPS spectra was performed using the least-squares method. The experimental data were deconvoluted by built-in software.

Powder X-ray diffraction (XRD) measurements were conducted at room temperature by using a Bruker D8 Advance (Bruker AXS,Karlsruhe,Germany) with Cu Ka radiation (0.154 nm, 40 kV, 40 mA). The interlayer spacing (d002) was calculated by the expression d_002_ = *n*/2sinθ (Bragg’s equation) [23].

Raman spectroscopy spectra were recorded from 600 to 2000 cm^−1^ on Raman spectrometer (Renishaw 2000) with λ = 514.5 nm to calculate the I_D_/I_G_ ratio (R) [24].

The overall morphologies of the B_0.30_-*o*-novolac were observed with a VEGA3 XMH scanning electron microscopy (SEM)(Tescan Co.,Brno,Czech Republic). Samples were measured after sputtering a thin layer of gold (1–2 nm).

## 3. Results and Discussion

### 3.1. Characterization of B-o-novolac and Cured B-o-novolac

**Figure 1:**
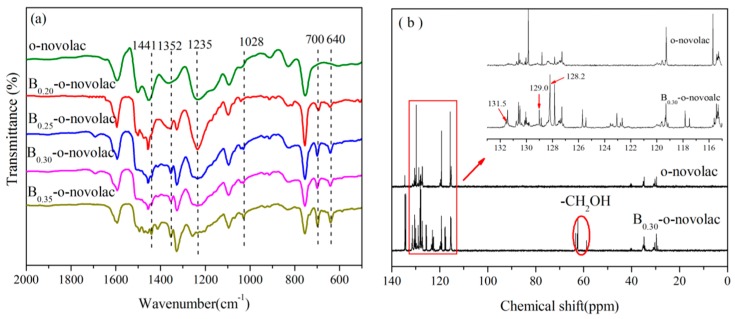
(**a**) FT-IR spectra for the B-*o*-novolac with different boron contents; (**b**) DEPT-135 spectra of novolac resins.

The structures of B-*o*-novolacs are confirmed via FT-IR, ^11^B NMR, and XPS. Figure 1a shows that the absorption bands assigned to the stretching vibration of B–O–C group appears at 1352 cm^−1^. The peak at 1235 cm^−1^ corresponds to the stretching vibration of phenolic hydroxyl C–O both in *o*-novolac and modified resin. With the increase of boric compound contents, the peak at 1235 cm^−1^ becomes weaker. The peak at 700 cm^−1^ is the stretching vibration of C–H in benzene ring in phenylboronic acid which has five adjacent H [10]. The peak at 1028 cm^−1^ is the unreacted hydroxymethyl groups in modified resin based on DFPT-135 spectra. The peak at 1441 cm^−1^ is assigned to the stretching vibration of B–O in phenylboronic acid and 640 cm^−1^ is the B–OH bending vibration which the *o*-novolac does not exist [25]. In Figure 1b, the peak at 63.5–58.8 ppm was assigned to the –CH_2_OH in B-*o*-navolac [11,22]. While for *o*-novolac, –CH_2_OH peaks (63.5–58.8 ppm) do not exist. Since boron is electron-deficient, peaks (115.1–114.9 ppm, 120.5–119.4 ppm) on the benzene ring linked to phenylboronic acid shift to low field. Peaks at 131.5, 129.0, and 128.2 ppm are assigned to the benzene ring in phenylboronic acid [26]. 

**Figure 2:**
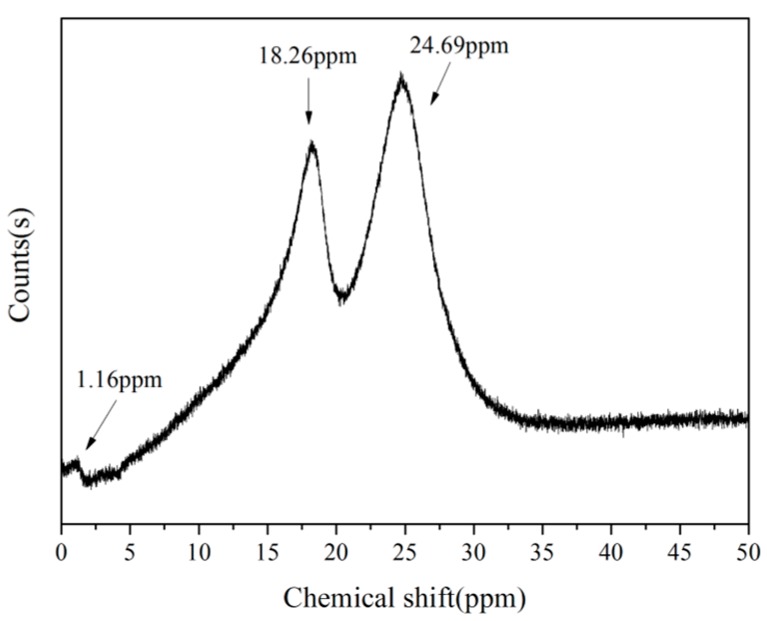
^11^B NMR spectrum of B_0.30_-*o*-novolac.

**Figure 3:**
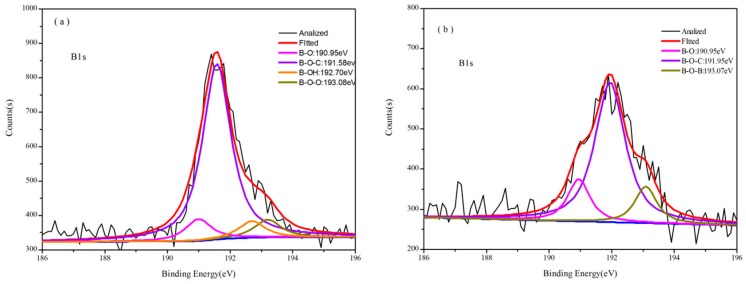

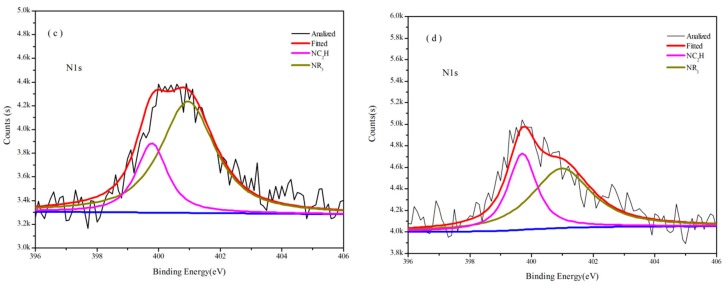
(**a**) B1s XPS spectra for B_0.30_-*o*-novolac; (**b**) B1s XPS spectra for cured B_0.30_-*o*-novolac; (**c**) N1s XPS spectra for cured B_0.30_-*o*-novolac; and (**d**) N1s XPS spectra for cured *o*-novolac.

In Figure 2, the ^11^B NMR spectrum of B_0.30_-*o*-novolac indicate that boron has at least three different compounds. The peak at 1.16 and 24.69 ppm are four-coordinate BO_4_ and boronates [22], respectively. The peak at 18.26 ppm is interpreted as the trigonal boron [27]. In Figure 3c, the boron elements are detected from the full-scan XPS spectrum of the B_0.30_-*o*-novolac. The B1s XPS spectrum can mainly be separated into four peaks at 190.95, 191.60, 192.70, and 193.20 eV [28,29] which are assigned to B–O, B–O–C, B–OH, and B–O–B. According to the study by Gao [19] *et al.*, the reaction activity of the B–OH with phenolic hydroxyl groups was lower than that with hydroxymethyl groups, and high temperature could increase reaction activity. In this test, when the concentration of boron in the reactoin mixture was increased, the possibility of the reaction at 140 °C between boric compounds and phenolic hydroxyl groups would increase according to the FT-IR in Figure 1. 

**Figure 4:**
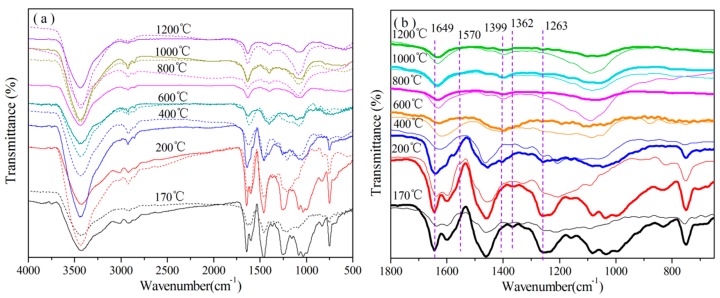
FT-IR for cured B_0.30_-*o*-novolac and cured *o*-novolac at different heat treatment temperatures (**a**) FT-IR for cured B_0.30_-*o*-novolac (the solid line) and cured *o*-novolac (dotted line) with a wavenumber ranging from 4000–500 cm^−1^; and (**b**) Figure 4a with a wavenumber ranging from 2000–650 cm^−1^ (B_0.30_-*o*- novolac: thick line; *o*-novolac: solid thin line).

**Figure 5:**
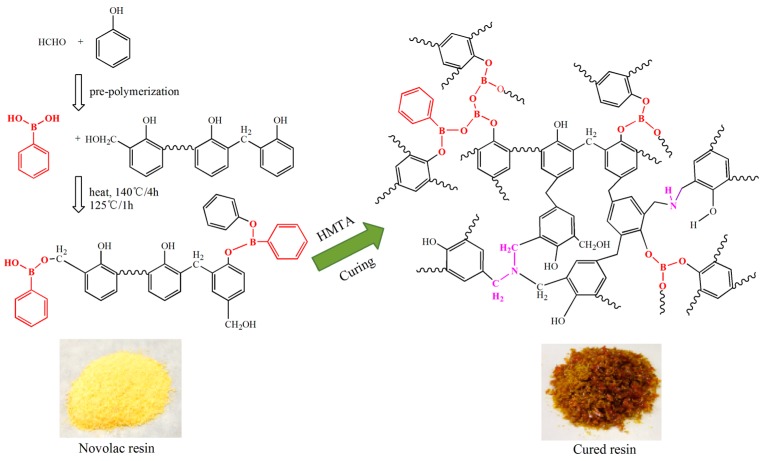
Schematic illustration of B-*o*-novolac synthetic and curing process.

The cured structures of B_0.30_-*o*-novolac and *o*-novolac were tested by XPS in Figure 3b–d and FT-IR in Figure 4. The absorption peak at 3415 cm^−1^ is attributed to the stretching vibration of N–H in NC_2_H which is overlapped with OH in phenol. The peak at 1649 and 1256 cm^−1^ are the deformation and stretching vibration of C–N, respectively [30,31]. The characteristic peaks (1441 and 640 cm^−1^) of B–OH disappeared after curing process. In Figure 3b, the B atom exists in at least three different chemical environments which could be attributed to B–O, B–O–C, and B–O–B types of chemical environment of B [16,22,28,32]. There are mainly two different chemical environments of N in both B-*o*-novolac and o-novolac, and can be assigned to the NR_3_ and NC_2_H as shown in Figure 5, corresponding to 401.00 ± 0.20 eV and 399.60 ± 0.20 eV [30,33,34] in Figure 3c,d.

### 3.2. Thermal Properties of B-o-novolacs

**Figure 6:**
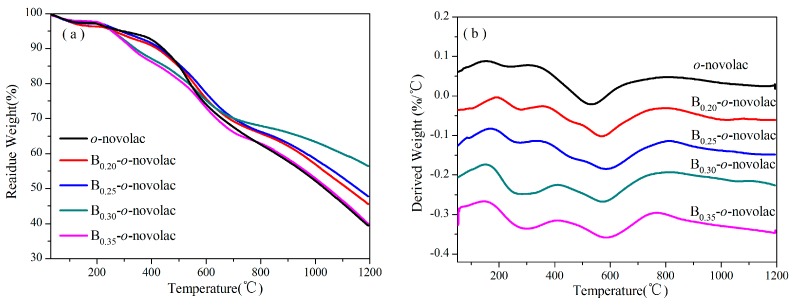
(**a**) TGA of B-*o*-novolacs from room temperature to 1200 °C with a heating rate of 10 °C/min under Ar atmosphere; (**b**) DTG of B-*o*-novolacs.

Thermal gravimetric (TGA) curves and derivative thermogravimetric (DTG) curves of the cured B-*o*-novolacs and *o*-novolac are shown in Figure 6. They are used to analyze the thermal stability of the resins. The representative thermal analysis data are listed in Table 1. The T_5%_ and T_10%_ are the decomposition temperature at 5% and 10% weight loss, whereas the maximum weight loss temperature (Tmax) is taken from the peak value of the DTG thermograms in Figure 6b. The TGA shows that char yields at 800 °C (C_800_) of cured B-*o*-novolacs modified with different boric compound contents are all higher than that of cured *o*-novolac. Except B_0.35_-*o*-novolac, C_800_ increases according to the increase of phenylboronic acids contents. After 1000 °C, the residual weights at 1000 °C (C_1000_) and at 1200 °C (C_1200_) decreased rapidly, especially for *o*-novolac. The C_1000_ and C_1200_ of B_0.30_-*o*-novolac are 63.34% and 56.41%, respectively, and 52.21% and 39.39% for cured *o*-novolac. The T_5%_ and T_10%_ of B-*o*-novolacs are lower than that of *o*-navolac, but the Tmax values are higher. And the weight losses of B-*o*-novolac increase with the increase of phenylboronic acid in the first stage (Table 1). When the content of phenylboronic acid is higher, some hydroxyl groups of phenylboronic acid not only reacted with hydroxyl groups in resins, but also with hydroxyl groups in phenol monomers at 140 °C. This would increase the phenol consumption and impede the chains to grow and leave some hydroxymethyl groups. The main products in this stage are H_2_O and CO. Except the condensation reaction involving phenolic hydroxyl groups and methyl groups, residual hydroxymethyl groups in B-*o*-novolacs (1028 cm^−1^ for –CH_2_OH vibration) [10] would react continuously to release water or methanol. Parts of B–C bonds would also break to remove benzene rings in stage 1 (stage 1:200 to 400 °C; stage 2:400 to 800 °C; stage 3:800 to 1000 °C) in DTG spectra [35,36,37,38,39]. These may be partial reasons why weight loss in stage 1 becomes higher with the increase of phenylboronic acid contents.

**Table 1:** TGA data of the cured *o*-novolac and B-*o*-novolacs.

Resin	T_5%_/°C	T_10%_/°C	T_max_/°C	C_800_/%	C_1000_/%	C_1200_/%	Weigh Loss in Stage 1 (%)
B_0.20_-*o*-novolac	260.93	419.61	568.82	65.76	56.94	45.54	5.39
B_0.25_-*o*-novolac	286.43	431.56	584.53	66.20	58.40	47.75	5.58
B_0.30_-*o*-novolac	255.02	340.05	574.86	67.89	63.34	56.41	10.01
B_0.35_-*o*-novolac	253.82	329.01	584.39	62.88	52.92	39.85	11.38
*o*-novolac	291.38	441.87	531.39	62.57	52.21	39.39	4.55

### 3.3. Chemical State of B and N during Pyrolysis Process

**Figure 7:**
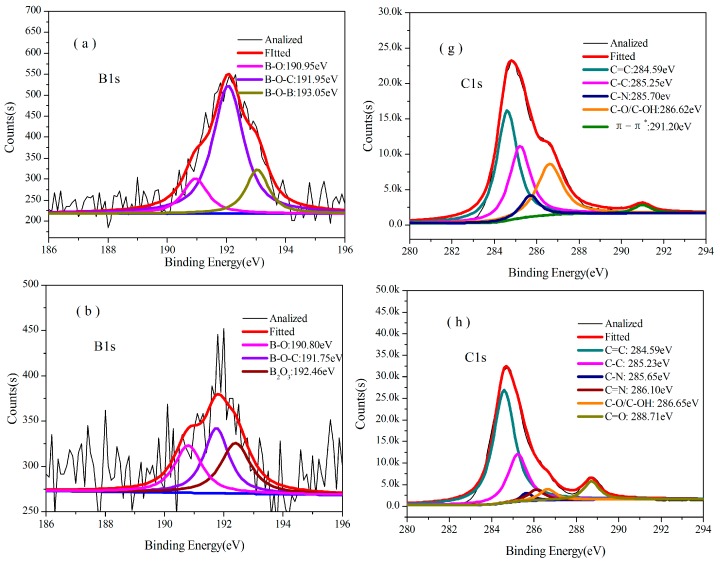

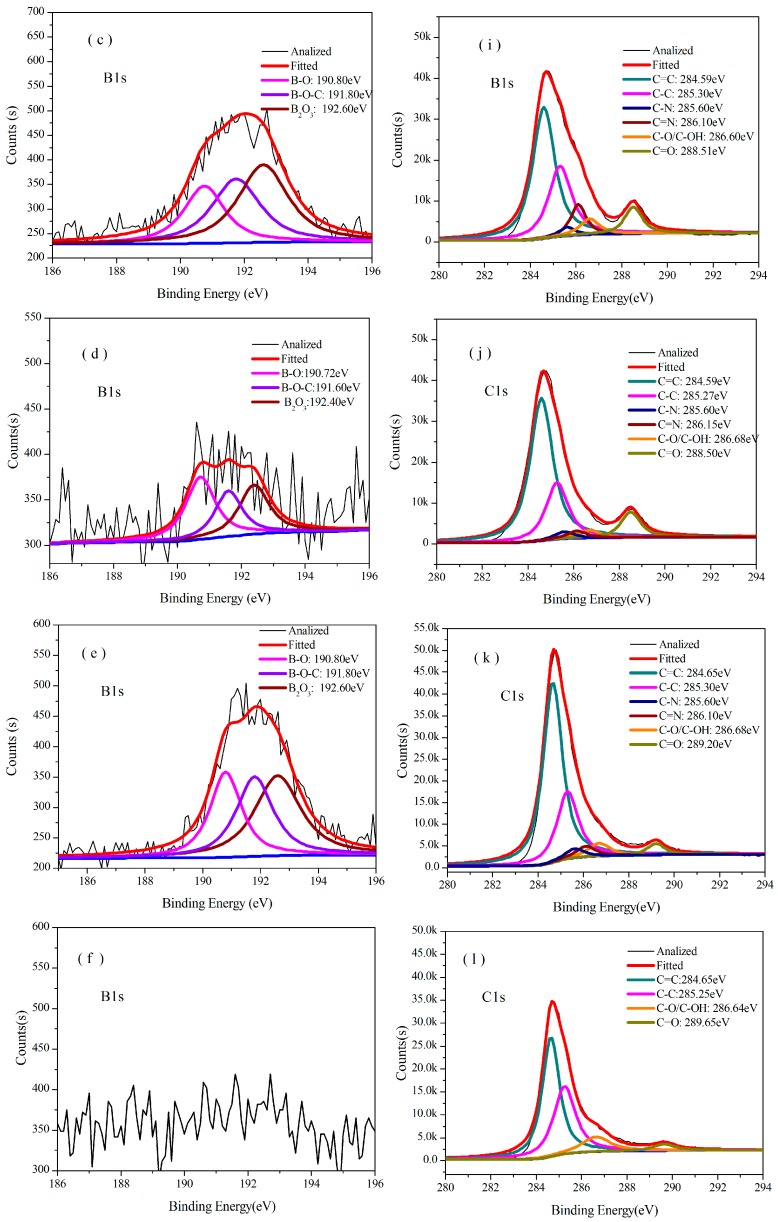
XPS spectra of B_0.30_-*o*-novolac at different pyrolysis temperatures. The deconvoluted B1s signal at (**a**) 200 °C; (**b**) 400 °C; (**c**) 600 °C; (**d**) 800 °C; (**e**) 1000 °C; (**f**) 1200 °C; C1s signal at (**g**) 200 °C; (**h**) 400 °C; (**i**) 600 °C; (**j**) 800 °C; (**k**) 1000 °C; and (**l**) 1200 °C.

**Figure 8:**
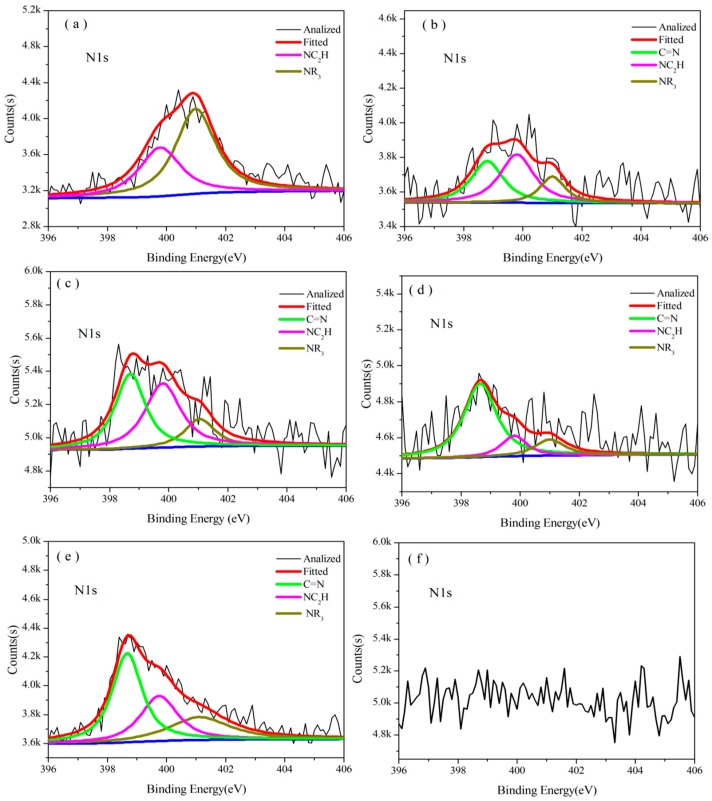
XPS spectra of B_0.30_-*o*-novolac at different pyrolysis temperatures. The deconvoluted N1s signal at (**a**) 200 °C; (**b**) 400 °C; (**c**) 600 °C; (**d**) 800 °C; (**e**) 1000 °C; and (**f**) 1200 °C.

**Figure 9:**
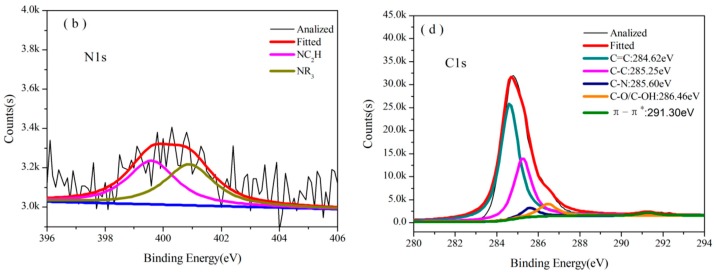
XPS spectra of *o*-novolac at different pyrolysis temperatures. The deconvoluted N1s signal at (**a**) 200 °C; (**b**) 400 °C; C1s signal at (**c**) 200 °C; and (**d**) 400 °C.

In order to confirm the presence and chemical states of B and N in the cured resins suffering from heating at different temperatures (200 °C/2 h, 400 °C/2 h, 600 °C/2 h, 800 °C/2 h, 1000 °C/2 h, and 1200 °C/2 h), FT-IR and core-level XPS measurements were taken as shown in Figure 4, Figure 7, Figure 8 and Figure 9. The effects of B and N on the pyrolysis process of modified resins are illustrated based on these tests.

#### 3.3.1. Analysis of FT-IR 

The FT-IR spectra of cured B_0.30_-*o*-novolac and cured *o*-novolac at different heating temperatures are shown in Figure 4. For cured B_0.30_-*o*-novolac at 200 °C, peaks at 1362 and 1399 cm^−1^ are assigned to B–O–C and B–O–B stretching vibrations.With increasing temperature, the intensity of peak B–O–C became weaker and B–O–B became stronger. Besides, the spectra in Figure 4b shows that the characteristic peak of benzene ring at 700 cm^−1^ decreased rapidly with the increasing of B–O–B vibration at 1399 cm^−1^.The bond energy of B–O and C–O were 516 and 326 kJ/mol, thus the formation of B–O–B structure in B_2_O_3_ was not only the cleavage of B–C bond, but also the O–C bonds, which is in accordance with the opinions of Wang’s groups [27,38,40,41,42]. Moreover, the dehydration reaction between two hydroxyl groups in phenylboronic acid or between phenylboronic acid and hydroxyl groups in resins in the curing process can also form B–O–B structure.

**Figure 10:**
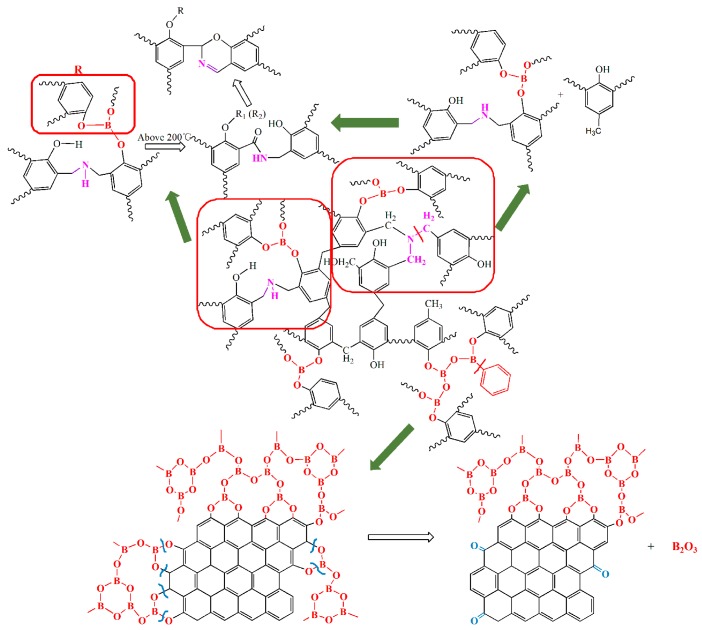
The possible mechanism of main chemical state changes of N and B in the pyrolysis process [28,48,49,50,51,52,53].

For the cured B_0.30_-*o*-novolac, when the heating temperature increased to 400 °C, the intensity of peak at 1256 cm^−1^ decreased and the peak at 1649–1599 cm^−1^ became stronger. It is because that the appearance of C=N stretching vibration, the peak at 1637 cm^−1^, is overlapped by the N–C structure and C=C structure in benzene ring [43]. The formation of C=N in six-member ring has been shown in Figure 10. As to *o*-novolac, the intensity of band at 1599–1640 cm^−1^ began to reduce above 200 °C.

#### 3.3.2. Analysis of XPS

Figure 7 presents B1s and C1s of XPS for B_0.30_-*o*-novolac at different heating temperatures. Figure 7a and Figure 3b have the same kinds of binding energy, which can be mainly resolved into three characteristic peaks, corresponding to B–O, B–O–C, and B–O–B. After treating at 400 °C for two hours (Figure 7b–e), the peak at 192.60 ± 0.20 eV representing B_2_O_3_ appeared [41]. In Figure 10, when the B_2_O_3_ formed as BO_3_ or BO_4_ units [44,45,46], the carbonyl groups also formed from the cleavage of O–C and B–O bonds. This could be the reason why the XPS of B-*o*-novolac existed in 288.50 eV peak and *o*-novolac did not have [47]. When the treating temperature increased to 1200 °C, boron almost disappeared.

Figure 8 shows the N1s XPS of B_0.30_-*o*-novolac treated at 200 °C/2 h, 400 °C/2 h, 600 °C/2 h, 800 °C/2 h, 1000 °C/2 h, and 1200 °C/2 h, and Figure 9 shows the N1s XPS of o-novolac treated at 200 °C/2 h and 400 °C/2 h, respectively. In Figure 8a, it is clear that N atom exists in mainly two different chemical environments that could be attributed to the presence of NR_3_ and NC_2_H, which is in accordance with the FT-IR in Figure 4 below 200 °C. When the treating temperature rose from 400 °C to 1000 °C, a new peak at 398.60 ± 0.20 eV in XPS (Figure 8b–e) appeared. This was assigned to C=N, which may form as shown in Figure 10 and could bear higher temperature [42,47,48,49]. 

Table 2 shows the contents of C, O, N, and B atoms of B_0.30_-*o*-novolac and C, O, and N of *o*-novolac at different treating temperatures. Table 3 shows the proportion of different chemical structures of N from N1s XPS of B_0.30_-*o*-novolac and *o*-novolac at different heating temperatures. The contents of C=N structure increased from 36.82% at 400 °C to 70.72% at 800 °C with the decreasing of NR_3_ and NC_2_H from 63.18% at 400 °C to 29.28% at 800 °C (Table 3). While for *o*-novolac, the XPS in Figure 9 shows that two individual lines are used with peaks centered at 401.00 ± 0.20 eV and 399.60 ± 0.20 eV which are assigned to NR_3_ and NC_2_H from 170 to 400 °C. With the treating temperature rising, the proportion of NC_2_H became larger than NR_3_. But after treating at 600 °C, the resin system rarely has N from the XPS measurement which was consistent with the research of Bertsch’s group [54]. So, from XPS analyses, N atoms in B_0.30_-*o*-novolac would slightly contribute to a higher char residual compared with *o*-novolac.

**Table 2:** The composition of the cured B_0.30_-*o*-novolac and its carbonization products obtained from the XPS analyses.

Temperature/°C	Atom ratio/%						
	Cured B_0.30_-*o*-novolac				Cured *o*-novolac		
	C	O	B	N	C	O	N
170	75.09	19.23	2.27	3.40	77.91	19.79	2.31
200	75.21	19.83	2.18	2.78	79.35	18.97	1.69
400	75.32	22.64	1.00	1.04	82.55	15.93	1.52
600	75.86	21.75	1.46	0.93	82.05	17.95	0
800	75.86	22.52	0.60	1.03	94.08	5.92	0
1,000	83.26	12.34	2.34	2.06	91.99	8.01	0
1,200	76.32	23.68	0	0	89.62	10.38	0

**Table 3:** The percentage area of the deconvoluted signals from XPS N1s of cured B_0.30_-*o*-novolac and o-novolac at different pyrolysis temperatures.

Temperature/°C	Resin					
	Component	Cured B_0.30_-*o*-novolac			Cured *o*-novolac	
		NR_3_	NC_2_H	C=N	NR_3_	NC_2_H
170	Binding Energy/eV	400.98	399.78	--	400.98	399.70
	Area/%	73.77	26.23	--	61.99	38.01
200	Binding Energy/eV	400.98	399.78	--	400.80	399.80
	Area/%	62.34	37.66	--	33.78	66.22
400	Binding Energy/eV	401.00	399.80	398.80	400.90	399.60
	Area/%	18.11	45.07	36.82	48.50	51.50
600	Binding Energy/eV	401.05	399.80	398.70	--	--
	Area/%	15.38	42.49	42.13	--	--
800	Binding Energy/eV	401.00	399.80	398.65	--	--
	Area/%	14.16	15.12	70.72	--	--
1,000	Binding Energy/eV	401.10	399.75	398.68	--	--
	Area/%	27.34	29.26	43.40	--	--

**Figure 11:**
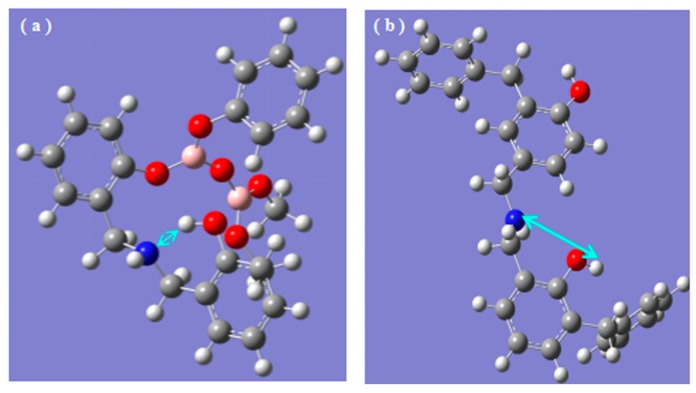
DFT: B3LYP/6-311G calculated structure of cured novolac resins: (**a**) cured B_0.30_-*o*-novolac; (**b**) cured *o*-novolac. C, gray; O, red; B, pink; N blue and the interaction force between N atom and H atom,light blue arrow.

In Figure 8 and Figure 9, nitrogen in cured B_0.30_-*o*-novolac would disappear after heating at 1200 °C, whereas at 600 °C for cured *o*-novolac. When pheneylboronic acid reacted with phenyl hydroxyl groups in the resin, formaldehyde would react at para sites and ortho active sites are left over because of steric effect. Thus, HMTA will react at ortho sites in the curing process. In order to study why N in cured modified resin could still be found at high temperature in comparison to cured *o*-novolac, the structures of modified resin and pure resin in Figure 11 were optimized by using the density functional theory (DFT) method B3LYP with 6-311G basis set in the Gaussian 09 program [55]. After curing, N of HMTA and H of phenol would form hydrogen bonding. The N⋯H distance of B_0.30_-*o*-novolac is 1.75 Å, which is shorter than that of *o*-novolac (4.29 Å) and the respective Wiberg bond index (WI) is 0.0973 which are larger than the WI of *o*-novolac (0.0002), implying that the interaction force of N⋯H in modified resin is stronger [56]. That is the reason why the deformation vibration peak of N–H of cured modified novolac in Figure 4b shifted to a lower wavenumber which was at 1570 cm^−1^ compared with o-novoalac. This is also the reason why N–H could keep in the system at higher temperature and form C=N structure above 200 °C.

#### 3.3.3. Analysis of SEM

**Figure 12:**
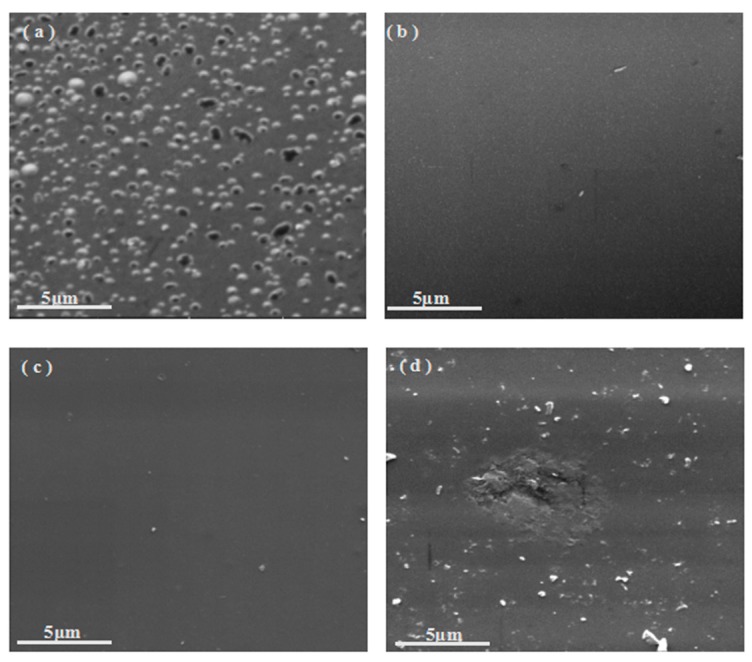
SEM of cured B_0.30_-*o*-novolac after treating at (**a**) 800 °C/2 h; (**c**) 1200 °C/2 h; SEM of cured o-novolac after treating at (**b**) 800 °C/2 h; and (**d**) 1200 °C/2 h in a tube furnace at a heating rate of 10 °C/min under a nitrogen atmosphere.

In Section 3.2, the weight masses of B_0.30_-*o*-novolac at 800 and 1200 °C are obviously higher than that of *o*-novolac. In order to illustrate the effect of B on the surface of cured resins, SEM images of B_0.30_-*o*-novolac and o-novolac treated at 800 and 1200 °C are compared in Figure 12. For cured B_0.30_-*o*-novolac in Figure 12a,c, it can be seen that some small particles which are B_2_O_3_ are on the surface of modified resin at 800 °C and they almost disappeared at 1200 °C with some voids. As for *o*-novolac in Figure 12b,d, there are some cavities on the surface at 800 °C. After treating at 1200 °C for 2 h, the surface becomes rougher compared with Figure 12c. The melting point of B_2_O_3_ is 450 °C, when the treating temperature is higher than 450 °C, B_2_O_3_ particles begin to melt, the volume expansion and wettability properties will help to cover the voids and defects on the surface of the cured resin, which preventing small molecules and carbon oxides from releasing [57]. Thus, the formation of B_2_O_3_ on the surface of resin can decrease the quantity of voids and contribute to the higher char yields. 

#### 3.3.4. Analysis of XRD

**Figure 13:**
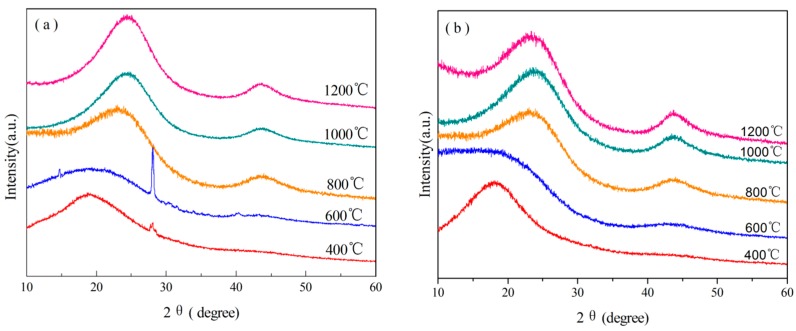
The XRD spectra of B_0.30_-*o*-novolac and *o*-novolac at different temperatures: (**a**) B_0.30_-*o*-novolac; and (**b**) *o*-novolac.

XRD analyses of B_0.30_-*o*-novolac heated at different temperatures were carried out to analyze the effects of B on the structural properties as shown in Figure 13. The XRD patterns of B modified resin in Figure 13a show the existence B at 400 and 600 °C. The peak at 28.06° can illustrate the formation of B_2_O_3_ starting at 400 °C [16,22,28]. Diffraction peaks of (002) and (100) are invisible in all XRD patterns when the treating temperature is above 600 °C. The (002) and (100) peaks are associated with the hexagonal graphite and the site and width may be different owing to the changeable interlayer spacing [58,59]. Due to the decrease of interlayer space., the (002) peak positions of B_0.30_-*o*-novolac are observed at 24.13°, 24.18°, and 24.50° at different heating temperatures, which are higher than that of *o*-novolac. Moreover, XRD patterns in Figure 13a show the sharper diffraction compared with Figure 13b. Table 4 lists the crystallite parameters of B_0.30_-*o*-novolac and *o*-novolac. As can be seen in Table 4, the d_002_ spacing decreases to 0.3603 nm with the increasing treating temperatures of B_0.30_-*o*-novolac. The d_002_ spacings of B_0.30_-*o*-novolac are all lower than that of *o*-novolac, indicating that B can improve the crystallization order of the materials [60,61].

**Table 4:** XRD and Raman spectra results of cured *o*-novolac and B_0.30_-*o*-novolac treated at different temperatures.

Resin	Temperature/°C	2θ(002)/°	d_002_-spacing/nm	D-band/cm^−1^	G-band/cm^−1^	R
*o*-novolac	800	23.06	0.3853	1,338	1,601	2.262
	1,000	23.65	0.3759	1,339	1,595	2.060
	1,200	23.38	0.3801	1,345	1,597	1.846
B_0.30_-*o*-novolac	800	24.13	0.3685	1,347	1,598	2.038
	1,000	24.18	0.3677	1,350	1,601	1.944
	1,200	24.50	0.3603	1,350	1,598	1.747

#### 3.3.5. Analysis of Raman Spectra

**Figure 14:**
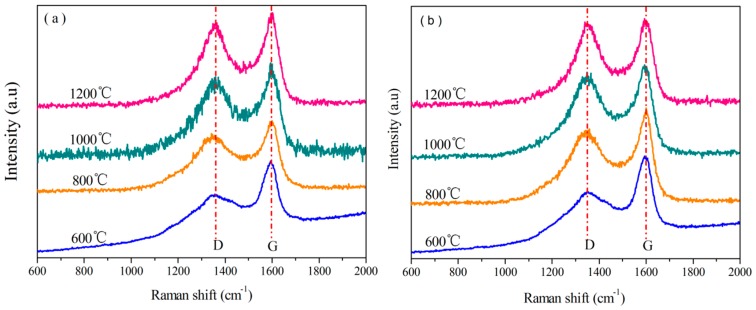
Raman spectra of B_0.30_-*o*-novolac and *o*-novolac heated at different temperatures: (**a**) B_0.30_-*o*-novolac; and (**b**) *o*-novolac.

Raman spectra were used to determine the effects of B on the carbon crystallite in B_0.30_-*o*-novolac (Figure 14). The results are shown in Table 4. There are two bands in the Raman spectra of B_0.30_-*o*-novolac and *o*-novolac. D-band located between 1338–1350 cm^−1^ is defect lattice vibration mode associated with disorder [62,63] and shows a small shift from the *o*-novolac to B_0.30_-*o*-novolac. G-band at about 1600 cm^−1^ representing graphite structure. The intensity of G-band elevates and D-band decreases slightly with the increasing treating temperature(Figure 14, Table 4). Moreover, the R of B_0.30_-*o*-novolac is lower than that of *o*-novolac at the same heating temperature. For B_0.30_-*o*-novolac, R decreases from 2.038 at 800 °C to 1.747 at 1200 °C, while the R for *o*-novolac is from 2.262 at 800 °C to 1.846 at 1200 °C. The changes in the Raman spectra show that introducing B into resins helps to decrease the disordered structure and to form graphite structure.

## 4. Conclusions

Modified *o*-novolacs are synthesized with different contents of phenylboronic acid. After introducing B into novolac resins the temperature of maximum decomposition rate increased by 43.5 °C, and the char yield increased by 5.3% at 800 °C, 11.13% at 1000 °C, and 17.02% at 1200 °C, respectively. Not only did the formation of B_2_O_3_ under pyrolysis contribute to the higher char yield of modified resin, but also the existence of nitrogen in the system. Due to hydrogen bonding of N and H, N could be found at higher temperature compared with cured *o*-novolac and stable C=N structure can be formed, which would reduce nitrogen loss. The formation of B_2_O_3_ from the cleavage of O–C and B–C at 400 °C helps to decrease the oxygen consumption, which may be the components of released gases, and the volume expansion of B_2_O_3_ above 450 °C in the system inhibits the volatilization of small molecules. Moreover, boron can improve the crystallinity and promote the graphite structure.

## Abbreviations

The following abbreviations are used in this manuscript:

HMTA: hexamethylenetetramine
DFT: Density functional theory
PF: Phenolic resin
BPF: boron-modified resin
B-*o*-novolac: boron-containing high ortho novolac resin
*o*-novolac: high ortho novalac resin
NMR: nuclear magnetic resonance
FT-IR: Fourier transform infrared
TGA: Thermalgravimetric analysis
XRD: X-ray diffraction
XPS: X-ray photoelectron spectroscopic

## References

[1] Choi M.H., Byun H.Y., Chung I.J. (2002). The effect of chain length of flexible diacid on morphology and mechanical property of modified phenolic resin. Polymer.

[2] Choi M.H., Chung I.J., Lee J.D. (2000). Morphology and curing behaviors of phenolic resin-layered silicate nanocomposites prepared by melt intercalation. Chem. Mater..

[3] Choi M.H., Jeon B.H., Chung I. (2000). The effect of coupling agent on electrical and mechanical properties of carbon fiber/phenolic resin composites. Polymer.

[4] Ma H., Wei G., Liu Y., Zhang X., Gao J., Huang F.T., Tan B., Song Z., Qiao J. (2005). Effect of elastomeric nanoparticles on properties of phenolic resin. Polymer.

[5] Cherian A.B., Varghese L.A., Thachil E.T. (2007). Epoxy-modified, unsaturated polyester hybrid networks. Eur. Polymer J..

[6] Chiang C.-L., Ma C.-C.M. (2004). Synthesis, characterization, thermal properties and flame retardance of novel phenolic resin/silica nanocomposites. Polymer Degrad. Stabil..

[7] Zhang Y., Lee S., Yoonessi M., Liang K., Pittman U.C. (2006). Phenolic resin-trisilanophenyl polyhedral oligomeric silsesquioxane (POSS) hybrid nanocomposites: Structure and properties. Polymer.

[8] Wang D.-C., Chang G.-W., Chen Y. (2008). Preparation and thermal stability of boron-containing phenolic resin/clay nanocomposites. Polymer Degrad. Stabil..

[9] Daniel C., Marianne B., Martin M. (2007). 100 years of bakelite, the material of a 1000 uses. Angew. Chem. Int. Edit..

[10] Ida P., Matjaž K. (2005). Characterization of phenol-formaldehyde prepolymer resins by in line FT-IR spectroscopy. Acta Chim. Slov..

[11] Mohamed O.A., Adriane L., Temisha M. (2003). Boron-modified phenolic resins for high performance applications. Polymer.

[12] Wang H., Guo Q., Yang J., Liu Z., Zhao Y., Lia J., Feng Z., Liua L. (2013). Microstructural evolution and oxidation resistance of polyacrylonitrile-based carbon fibers doped with boron by the decomposition of B_4_C. Carbon.

[13] Wang J., Jiang N., Jiang H. (2010). Micro-structural evolution of phenol-formaldehyde resin modified by boron-carbide at elevated temperatures. Mater. Chem. Phys..

[14] Wang J., Guo Q., Liu L., Song J. (2005). The preparation and performance of high-temperature adhesives for graphite bonding. Int. J. Adhes. Adhes..

[15] Wang J., Jiang N., Jiang H. (2009). Effect of the evolution of phenol-formaldehyde resin on the high-temperature bonding. Int. J. Adhes. Adhes..

[16] Wang S., Jing X., Wang Y., Si J. (2014). High char yield of aryl boron-containing phenolic resins: The effect of phenylboronic acid on the thermal stability and carbonization of phenolic resins. Polymer Degrad. Stabil..

[17] Cameron G.C., James E. (1995). Oxidative and hydrolytic stability of boron nitride-a new approach to improving the oxidation resistance of carbonaceous structures. Carbon.

[18] Gao J., Jiang C., Su X. (2010). Synthesis and thermal properties of boron-nitrogen containing phenol formaldehyde resin/MMT nanocomposites. Int. J. Polymer Mater..

[19] Gao J., Liu Y., Yang L. (1999). Thermal stability of boron-containing phenol formaldehyde resin. Polymer Degrad. Stabil..

[20] Fan D.B., Li G.Y., Qin T.F., Chu F.-X. (2014). Synthesis and structure characterization of phenol-urea-formaldehyde resins in the presence of magnesium oxide as catalyst. Polymers.

[21] Hu Y., Geng W., You H., Wang Y., Loy D.A. (2014). Modification of a phenolic resin with eoxy- and methacrylate-functionalized silica sols to improve the ablation resistance of their glass fiber-reinforced composites. Polymers.

[22] Wang S., Jing X., Wang Y. (2014). Synthesis and characterization of novel phenolic resins containing aryl-boron backbone and their utilization in polymeric composites with improved thermal and mechanical properties. Polymer Adv. Technol..

[23] Endo M., Kim C., Karaki T., Nishimura Y., Matthews M.J., Brown S.D.M., Dresselhaus M.S. (1998). Structural analysis of the B-doped mesophase pitch-based graphite fibers by Raman spectroscopy. Phys. Rev. B.

[24] Tuinstra F., Koening J.L. (1970). Ranman spectrum of graphite. J. Chem. Phys..

[25] Gouin X., Grange P., Bois L. (1995). Characterization of the nitridation process of phenylboronic acid. J. Alloys Compd..

[26] Odom J.D., Moore T.F. (1979). Nuclear magnetic resonance studies of boron compounds: XVI. Carbon-13 studies of organoboranes: Phenylboranes and boron-substituted aromatic heterocycles. J. Organomet. Chem..

[27] Joseph W.E., Weiss D.L.B. (2010). A solid-State 11B NMR and computational study of boron electric field gradient and chemical shift tensors in boronic acids and boronic esters. J. Phys. Chem. A.

[28] Wang S., Wang Y., Bian C., Zhonga Y., Jing X. (2015). The thermal stability and pyrolysis mechanism of boron-containing phenolic resins: The effect of phenyl borates on the char formation. Appl. Surf. Sci..

[29] Bois L., L’Haridon P., Laurenta Y., Gouin X., Grange P., Létard J.-F., Birot M., Pillot J.-P., Dunoguès J. (1996). Characterization of a boro-silicon oxynitride prepared by thermal nitridation of a polyborosiloxane. J. Alloys Compd..

[30] Wang S., Wang Y., Bian C. (2015). Effects of chemical structure and cross-link density on the heat resistance of phenolic rensin. Polymer Degrad. Stabil..

[31] Wang D., Li B., Zhang Y., Lu Z. (2013). Triazine-containing benzoxazine and its high-performance polymer. J. Appl. Polymer Sci..

[32] Burgess J.S., Acharya C.K., Lizarazo J., Yancey N., Flowers B., Kwon G., Klein T., Weaver M., Lane A.M., Turnerb C.H. (2008). Boron-doped carbon powders formed at 1000 °C and one atmosphere. Carbon.

[33] Wang B., Huang Y., Liu L. (2006). Effect of solvents on adsorption of phenolic resin onto -aminopropyl-triethoxysilane treated silica fiber during resin transfermolding. J. Mater. Sci..

[34] Iwazaki T., Obinata R., Sugimoto W. (2009). High oxygen-reduction activity of silk-derived activated carbon. Electrochem. Commun..

[35] Jackson W.M., Conley R.T. (1964). High temperature oxidative degradation of phenol-formaldehyde polycondensates. J. Appl. Polym. Sci..

[36] Bessire B.K., Lahankar S.A., Minton T.K. (2015). Pyrolysis of phenolic impregnated carbon ablator. ACS Appl. Mater. Inter..

[37] Thilagar P., Murillo D., Chen J., Jäkle F. (2013). Synthesis and supramolecular assembly of the bifunctional borinic acid [1, 2-fcB(OH)]_2_. Dalton Trans..

[38] Jiang H., Wang J., Wu S., Yuan Z., Hu Z., Wu R., Liu Q. (2012). The pyrolysis mechanism of phenol formaldehyde resin. Polym. Degrad. Stabil..

[39] Zhao Y., Yan N., Feng M.W. (2013). Thermal degradation characteristics of phenol–formaldehyde resins derived from beetle infested pine barks. Thermochim. Acta.

[40] Hall D.G. (2012). Boronic Acids: Preparation and Applications in Organic Synthesis, Medicine and Materials.

[41] William C., Thomas E.P., Carina O. (1995). Synthesis and characterization of boron-doped carbons. Carbon.

[42] Ardelean P.P. (2004). Comparative vibrational study of *x*Fe_2_O_3_(1−*x*) [3B_2_O_3_MO](MOCaO or CaF_2_) glass systems. Mater. Lett..

[43] Cheng Y., Yang J., Jin Y., Deng D., Xiao F. (2012). Synthesis and properties of highly cross-linked thermosetting resins of benzocyclobutene-functionalized benzoxa-zine. Macromolecules.

[44] Kashiwakura S., Takahashi T., Maekawa H., Tetsuya N. (2010). Applica-tion of ^11^B MAS-NMR to the characterization of boron in coal fly ash generated from Nantun coal. Fuel.

[45] Anja W.-B., Doris M., Dimitrios P. (2015). Structure and properties of orthoborate glasses in the Eu_2_O_3_-(Sr,Eu)O-B_2_O_3_ quaternary. J. Phys. Chem. B.

[46] Wu H., Wei Q., He H., Yang B.-F., Zhang Q., Yang G.-Y. (2014). A new acentric metal borate Mg [B_6_O_9_(OH)_2_]·4H_2_O: Synthesis, structure and optical property. Inorg. Chem. Commun..

[47] Meng L., Zhang X., Tang Y., Su K., Kong J. (2015). Hierarchically porous silicon-carbon-nitrogen hybrid materials towards highly efficient and selective adsorption of organic dyes. Sci. Rep..

[48] Agag T., Liu J., Graf R., Spiess H.W., Ishida H. (2012). Benzoxazole resin: A novel class of thermoset polymer via smart benzoxazine resin. Macromolecules.

[49] Kim S.-K., Choi S.-W., Jeon W.S., Park J.O., Ko T., Chang H. (2012). Cross-linked benzoxazine-benzimidazole copolymer electrolyte membranes for fuel cells at elevated temperature. Macromolecules.

[50] Ashourirad B., Sekizkardes A.K., Altarawneh S. (2015). Exceptional gas adsorption properties by nitrogen-doped porous carbons derived from benzimidazole-linked polymers. Chem. Mater..

[51] Guo J., Rao Q., Xu Z. (2010). Effects of particle size of fiberglass-resin powder from PCBs on the properties and volatile behavior of phenolic molding compound. J. Hazard. Mater..

[52] Zhang X., Mark G.L., David H.S. (1997). The chemistry of novolac resins: 3. ^13^C and ^15^N n.m.r, studies of curing with hexamethylenetetramine. Polymer.

[53] Zhang X., Alan C.P., David H.S. (1998). The chemistry of novolac resins-V. Reactions of benzoxazine intermediates. Polymer.

[54] Lytle C.A., Bertsch W., McKinley M. (1998). Determination of novolac resin thermal decomposition products by pyrolysis-gas chromatography-mass spectrometry. J. Anal. Appl. Pyrol..

[55] Costa A.C., Ramos J.M., Téllez Soto C.A., Martin A.A., Raniero L., Ondar G.F., Versiane O., Moraes L.S. (2013). Fourier transform infrared and raman spectra, DFT:B3LYP/6–311G(d,p) calculations and structural properties of bis(diethyldithiocarbamate) copper(II). Spectrochim. Acta A.

[56] Serpell Christopher J., Kilah Nathan L., Costa Paulo J., Felix V., Beer P.D. (2010). Halogen bond anion templated assembly of an imidazolium pseudorotaxane. Angew. Chem. Int. Edit..

[57] Wu X., Radovic R.L. (2005). Inhibition of catalytic oxidation of carbon/carbon composites by boron-doping. Carbon.

[58] Norio I., Chong R.P., Hiroyuki F. (2004). Specification for a standard procedure of X-ray diffraction measurements on carbon materials. Carbon.

[59] Lyu S.C., Han J.H., Shin K.W. (2011). Synthesis of boron-doped double-walled carbon nanotubes by the catalytic decomposition of tetrahydrofuran and triisopropyl borate. Carbon.

[60] Wang H., Han T., Yang J., Tao Z., Guo Q., Liu Z., Feng Z., Liu L. (2014). Structural evolution of rayon-based carbon fibers induced by doping boron. RSC Adv..

[61] Zhong D.H., Sano H., Uchiyama Y., Kobayashi K. (2000). Effect of low-level boron doping on oxidation behavior of polyimide-derived carbon films. Carbon.

[62] Matthews M.J., PimentM A., Dresselhaus G. (1999). Origin of dispersive effects of the Raman D band in carbon materials. Phys. Rev. B.

[63] Nikiel L., Jagodzinski P.W. (1993). Raman spectroscopic characterization of graphites: A re-evalution of spectra/structure correlation. Carbon.

